# Evolution of Cooperation in Social Dilemmas on Complex Networks

**DOI:** 10.1371/journal.pcbi.1004779

**Published:** 2016-02-29

**Authors:** Swami Iyer, Timothy Killingback

**Affiliations:** 1 Computer Science Department, University of Massachusetts, Boston, Massachusetts, United States of America; 2 Mathematics Department, University of Massachusetts, Boston, Massachusetts, United States of America; ETH Zurich, SWITZERLAND

## Abstract

Cooperation in social dilemmas is essential for the functioning of systems at multiple levels of complexity, from the simplest biological organisms to the most sophisticated human societies. Cooperation, although widespread, is fundamentally challenging to explain evolutionarily, since natural selection typically favors selfish behavior which is not socially optimal. Here we study the evolution of cooperation in three exemplars of key social dilemmas, representing the prisoner’s dilemma, hawk-dove and coordination classes of games, in structured populations defined by complex networks. Using individual-based simulations of the games on model and empirical networks, we give a detailed comparative study of the effects of the structural properties of a network, such as its average degree, variance in degree distribution, clustering coefficient, and assortativity coefficient, on the promotion of cooperative behavior in all three classes of games.

## Author Summary

Social dilemmas embody the tension between individual self-interest on the one hand and the public good on the other that underlie many of the greatest challenges facing human and animal societies, such as the maintenance of altruism and the responsible use of common resources. Understanding the mechanisms through which cooperative, socially optimal, behavior can be established in social dilemmas is a fundamental problem in evolutionary biology and in many areas of the social sciences. Here we study how cooperative behavior can emerge in three key social dilemmas—known as the donation game, snowdrift game and sculling game—when interactions between individuals form a network. We show that in all three social dilemmas significantly higher levels of cooperative behavior typically emerge in such a situation as compared to what would be the case in the absence of any network structure. In particular, we show that certain structural properties that are common in real-world social networks have a significant effect in increasing the level of cooperative behavior that is present in all three social dilemmas.

## Introduction

The evolution and maintenance of cooperation in social dilemmas is essential for the formation of systems at all levels of complexity, from replicating molecules to multi-cellular organisms to human societies. A small selection of examples of cooperative behavior in social dilemmas from the bewilderingly large number of possible instances include: assembly of the earliest replicating molecules to form larger replicating entities capable of encoding more information [1, 2]; integration of once free-living prokaryote ancestors of mitochondria and chloroplasts into eukaryotic cells [2]; differential production of intracellular products needed for replication in an RNA phage [3]; vampire bats donating blood meals to roost mates [4]; predator inspection in sticklebacks and guppies [5]; allogrooming in social animals [6]; alarm calls by mammals and birds in response to danger [7]; contribution to providing public goods [8], such as social security programs; restraint in the consumption of common resources [9, 10], including responsible use of fishing grounds, limiting the emission of pollution into the atmosphere, and sharing Internet bandwidth; correct implementation of the TCP protocol so as to avoid congestion in Internet traffic [11]; and sharing files over a peer-to-peer network [12].

In spite of the ubiquity of cooperation in social dilemmas, achieving a satisfactory understanding of the origin and stability of this phenomenon is fundamentally difficult [2, 13–18]. This difficulty resides in the very nature of a social dilemma, which in classical game theory may be defined as a game which possesses at least one socially inefficient Nash equilibrium [16, 19]. At this Nash equilibrium there is no incentive for any individual to change their behavior, and yet because it is socially inefficient there is at least one other outcome in which all individuals would be better of. Here we shall be concerned with the notion of a social dilemma that arises in evolution, which we shall define analogously to be a game which possesses at least one socially inefficient evolutionary attractor. Such an evolutionary attractor could be, for example, an evolutionary stable strategy [20, 21], a stable equilibrium point of the replicator dynamics [22, 23] (where, in fact, the former implies the latter), or an attracting state in stochastic evolutionary dynamics [24–29]. In such a social dilemma, adopting the strategy at the socially inefficient attractor is considered to be defection, while adopting the socially efficient strategy is taken to be cooperation. The nature of the dilemma is now apparent—individuals employing strategies corresponding to the socially inefficient attractor will be trapped there by natural selection, in spite of all individuals being better off if they could adopt socially efficient behavior.

In this paper we study the evolution of cooperation in three symmetric, two-player, two-strategy (i.e., symmetric 2 × 2) games, which are exemplars of social dilemmas in the three fundamental classes of symmetric 2 × 2 games—the prisoner’s dilemma, hawk-dove, and coordination classes.

The first game we consider is the donation game, which is the fundamental exemplar in the prisoner’s dilemma class of games, and provides the basic game theory model for altruism [13, 17, 18]. The second game, the snowdrift game, is an exemplar of a social dilemma in the hawk-dove class of games [15, 18]. While games of hawk-dove type have been extensively studied as models of conflicts and contests [20, 21, 30], the snowdrift game provides an interesting model for certain types of cooperative behavior that differ from pure altruism [15, 17]. The third game that we introduce and study here, which we call the sculling game, is an exemplar of a social dilemma in the coordination class of games. Games in the coordination class have been widely used as models for conventions [24, 25, 31], but they have typically received little attention as models of cooperation, although interesting exceptions to this trend are [32–35]. The sculling game, as we define it, is an exemplar of a game in the coordination class in an analogous sense to that in which the snowdrift game is an exemplar in the hawk-dove class of games. We introduce the sculling game with the intention of using it as a model for certain types of cooperative behavior not described by the donation or snowdrift games. It is worth remarking that in [2] two games were mentioned very briefly in passing, which were referred to as the “rowing game” and the “sculling game”, however, these games are unrelated to the sculling game that we introduce here (in [2] the “rowing game” is a coordination game and the “sculling game” is a prisoner’s dilemma game).

A number of different approaches to understanding the evolution of cooperation in social dilemmas have been studied [36]. These include: kin selection [13], direct reciprocity [14, 37–39], indirect reciprocity [40, 41], evolution in network-structured populations [42–50], and evolution in group structured populations [51, 52].

In this paper we study the evolutionary dynamics of the donation, snowdrift, and sculling games in structured populations modeled by complex networks, where vertices represent individuals in the population and edges between vertices indicate that the corresponding individuals may interact. We use individual-based simulations to investigate the evolutionary dynamics of the three games on a variety of model and empirical networks, and we give a detailed comparative study of the effects of network properties, such as average degree, variance in degree distribution, clustering coefficient, and assortativity coefficient, on the promotion of cooperative behavior in all of these games.

There is an extensive literature concerned with the evolution of cooperation in structured populations, which we will briefly review so as to put the current work in context. Many studies have focused on understanding how cooperation arises in spatially structured populations which can be modeled by regular lattices. A large majority of these works have studied the evolution of cooperation in the prisoner’s dilemma game or variants of it [42, 43, 45, 47–50, 53–60]. A smaller number of studies of lattice structured populations have focused on other games such as the hawk-dove game or the snowdrift game [44, 46, 61, 62]. More recently there has been a shift of attention towards studying the evolution of cooperation in structured populations that are modeled by complex networks [63–66]. Again the vast majority of these investigations have focused on studying various versions of the prisoner’s dilemma game [47–49, 67–73], but with some studies also on other games such as the snowdrift game [48, 74–76] or stag hunt game [34, 35].

Recent work has also focussed on other interesting aspects of the evolution of cooperation in structured populations, including: the use of effective payoffs in the prisoner’s dilemma game [77]; the effect of random and targeted removal of vertices [78]; cooperation in populations with multiple interaction layers [79]; the effect of social influence [80]; the consequences of dynamical linking [81, 82]; and the evolution of cooperation in other games, such as the traveler’s dilemma game and the minimum effort coordination game [19, 83].

No brief overview of the literature on the evolution of cooperation in structured populations can do justice to the enormous body of work on this subject, and for more detailed discussions we recommend that the interested reader consult one or more of the excellent reviews available [50, 84–87].

The main conclusion that emerges from previous studies is that for the prisoner’s dilemma game and its variants structured populations allow cooperation to be maintained under suitable circumstances, which is never possible in a well-mixed population [42, 43, 45, 47–49, 53–55, 57–60, 67–73]. For the hawk-dove game and the snowdrift game the results are less clear-cut. For the hawk-dove game, lattice structured populations typically result in increased levels of cooperation [44], however, for the snowdrift game, cooperation may be inhibited [46] or promoted [48] by different population structures. The small number of studies of the stag hunt game suggest that structured populations can also promote the evolution of cooperation in this game [34, 35].

Despite the large number of studies on the evolution of cooperation in structured populations, surprisingly little is known about how the fundamental properties of a network structured population, such as mean degree, heterogeneity in degree distribution, clustering and assortativity, affect the evolution of cooperation. What little is known in this direction is almost completely confined to the effect of average network degree on the evolution of cooperation in the prisoner’s dilemma game [49, 67], with almost nothing being known about the effect of other structural properties of networks on the evolution of cooperation in the prisoner’s dilemma game or of the effect of almost any structural network properties on the emergence and maintenance of cooperation in games in the hawk-dove or coordination classes. A further limitation of previous studies of cooperation in network structured populations is that there is typically no clear way to compare the levels of cooperation that arise in games representing different classes of social dilemmas. For example, there is no straightforward way to compare the level of cooperation that occurs in the prisoner’s dilemma game [42, 43, 45, 47–49, 53–55, 57–60, 67–73] with those that occur in the snowdrift game [46] or the stag-hunt game [34, 35]. As a result of this lack of comparability, previous work on the evolution of cooperation in structured populations has led to many isolated results, but much less in the way of general understanding that applies across a wide range of social dilemmas.

The purpose of this paper is to provide a systematic and comparative study of the evolution of cooperation for those games which serve as canonical representatives of the three fundamental classes of symmetric 2 × 2 games—the prisoner’s dilemma, hawk-dove, and coordination classes of games—in a wide variety of structured populations. The three canonical games that we study here are the donation game, the snowdrift game and the sculling game, which provide exemplars of the the prisoner’s dilemma, hawk-dove, and coordination classes of games, and with which the evolution of cooperation in all three classes of games in structured populations can be studied in a uniform way.

Aspects of the evolution of cooperation in structured populations have previously been studied for the donation game in [49] and for the snowdrift game in [46, 48]. The sculling game is introduced for the first time here, with the aim of providing a canonical representative game in the coordination class of games that can be used to study cooperative phenomena. We regard the sculling game as playing a similar role in the coordination class of games to that played by the snowdrift game in the hawk-dove class of games, and hope that the introduction of this game will stimulate further study of cooperative behavior in coordination-type games.

The major theme of this paper is to understand how the fundamental structural properties of a network affect the evolution of cooperation in the donation, snowdrift and sculling games. For complex networks the key structural properties include [63–66]: mean degree, heterogeneity of the degree distribution, clustering coefficient, and assortativity coefficient. As noted above, most previous work on cooperation in structured populations has centered on the prisoner’s dilemma game and its variants, but even in this case little is known about the effect of the structural properties of the network on the evolution of cooperation. Still less is known in this direction for the snowdrift and stag hunt games. Determining how the level of cooperation in each of these three games depends on the structural properties of the network, and comparing the levels of cooperation that arise in different games, is a non-trivial problem even in principle, since the level of cooperation that emerges in a particular game on a given network will depend on the parameters that enter into the definition of the game. To overcome this difficulty, we show that for the donation, snowdrift and sculling games it is possible to define a single integrated measure of the total degree of cooperation in the game on a network which is independent of the parameters specifying the game. This measure, which we refer to as the *cooperation index*, allows the total degree of cooperation in all three games on a network—and, crucially, the dependence of the total degree of cooperation on the structural properties of the network—to be unambiguously computed and compared. These developments represent the major novelty of the present work—we determine in a systematic and uniform fashion, which allows direct comparison, the degree of cooperation that evolves in the donation, snowdrift and sculling games on a wide variety of model and empirical networks, and further study how the level of cooperation in these games depends on the fundamental structural properties of the networks.

## Models

### Exemplars of Social Dilemmas

In this section we define the three symmetric 2×2 games that we use to model the evolution of cooperation. These games are exemplars of the prisoner’s dilemma, hawk-dove and coordination classes of games, which we will refer to as the donation, snowdrift and sculling games, respectively.

The payoff matrix of a symmetric 2 × 2 game, with strategies *C* (cooperation) and *D* (defection) is given by:

$$\pi =\begin{array}{cc} & \begin{array}{cc} C & D \end{array}\\ \begin{array}{c} C\\ D \end{array} & \left[\begin{array}{cc} \alpha & \beta \\ \gamma & \delta \end{array}\right] \end{array},$$ (1)

where α,β,γ,δ∈R. The specific values of the elements of the payoff matrix depend on the game under consideration.

Consider a large, well-mixed population of individuals playing the symmetric 2 × 2 game with payoff matrix given by matrix 1. Let *p* denote the frequency of individuals playing strategy *C*, with frequency 1 − *p* playing strategy *D*. The evolutionary dynamics of the population is governed by the replicator equation [17, 22, 23]

$$\begin{array}{ll} \dot{p} & =p\left(f_{C}-\bar{f}\right)\\ & =p\left(1-p\right)\left[p\left(\alpha -\gamma \right)+\left(1-p\right)\left(\beta -\delta \right)\right], \end{array}$$ (2)

where *f*_*C*_ = *pπ*(*C*, *C*) + (1 − *p*)*π*(*C*, *D*) and *f*_*D*_ = *pπ*(*D*, *C*) + (1 − *p*)*π*(*D*, *D*) denote the fitnesses of the strategies *C* and *D*, respectively, and $\bar{f}=pf_{C}+\left(1-p\right)f_{D}$ denotes the mean fitness of the population.

#### Donation game

The donation game is the fundamental exemplar in the prisoner’s dilemma class of games, and provides the basic game theory model for altruism [13, 17, 18]. The origin of the game can be seen in the following scenario. Suppose that the act of donating blood to someone incurs a cost *c* to the donor but confers a benefit *b* to the recipient, where $b,c\in R_{+}$ and *b* > *c*. Such a situation could arise, for example, if the donor produces an important blood factor that the recipient does not produce. Consider two individuals, John and Bill, each of whom produces such a blood factor that the other lacks. If both donate and receive blood, then each obtains a payoff of *b* − *c*. If neither donates nor receives blood, then both obtain a payoff of 0. If one donates but does not receive blood then the donor obtains a payoff of − *c*, and, similarly, if one receives but does not donate blood, then the recipient obtains a payoff of *b*. If we consider donation as the cooperative strategy *C* and non-donation as the defective strategy *D*, then the scenario described above can be modeled as a symmetric 2 × 2 game, called the *donation game*, with payoff matrix:

$$\pi =\begin{array}{cc} & \begin{array}{cc} C & D \end{array}\\ \begin{array}{c} C\\ D \end{array} & \left[\begin{array}{cc} b-c & -c\\ b & 0 \end{array}\right] \end{array}.$$ (3)

It is clear from eq 2 that the replicator equation for any symmetric 2 × 2 game is invariant under an affine transformation of the payoff matrix 1, modulo a rescaling of the time variable. Thus, the evolutionary dynamics of any game with payoff matrix 1 is unchanged by an affine transformation of the payoff matrix. Therefore, we can divide each element of the above matrix by *b* and denote the cost-to-benefit ratio $\frac{c}{b}$ by *ρ* to obtain the following equivalent payoff matrix for the donation game:

$$\pi =\begin{array}{cc} & \begin{array}{cc} C & D \end{array}\\ \begin{array}{c} C\\ D \end{array} & \left[\begin{array}{cc} 1-\rho & -\rho \\ 1 & 0 \end{array}\right] \end{array},$$ (4)

where *ρ* ∈ (0, 1). We note that it follows directly from the rank ordering of the elements of the payoff matrix 4 that the donation game is in the prisoner’s dilemma class of symmetric 2 × 2 games.

For the donation game with payoff matrix 4, the unique Nash equilibrium is (*D*, *D*), which is also the unique evolutionarily stable strategy (ESS). Eq 2 gives the evolutionary dynamics for the game to be $\dot{p}=-p\left(1-p\right)\rho$. The equilibrium points for the dynamics are *p*^⋆^ = 0 and *p*^⋆^ = 1, the former being asymptotically stable, while the latter is unstable. Thus, a population of individuals playing the donation game, starting from an initial frequency *p*_0_ ∈ (0, 1) of cooperators, will evolve towards the all-defector (*p*^⋆^ = 0) equilibrium state.

#### Snowdrift game

The snowdrift game is an interesting exemplar of a social dilemma in the hawk-dove class of games, and provides a model for certain types of cooperative behavior that differ from pure altruism [15, 17, 18]. The form of this game is typically introduced through the following story. Two individuals John and Bill are stuck in a car on their way home because the road is blocked by a snowdrift. Each individual can either get out of the car and shovel to clear the snow or stay in the car and not shovel. Let the benefit of getting home be *b* and the cost of clearing the snow be *c*, where $b,c\in R_{+}$ and *b* > *c*. If both John and Bill shovel, then the amount of work for each is halved, so each of them gets a payoff of $b-\frac{c}{2}$. If neither John nor Bill shovels, then they both remain stuck in the snowdrift and both receive a payoff of 0. If one shovels and the other does not, then the payoff to the shoveler is *b* − *c* while the payoff to the non-shoveler is *b*. If we consider shoveling as the cooperative strategy *C* and non-shoveling as the defective strategy *D*, then we can model the scenario described above as a symmetric 2 × 2 game, called the *snowdrift game*, with the payoff matrix

$$\pi =\begin{array}{cc} & \begin{array}{cc} C & D \end{array}\\ \begin{array}{c} C\\ D \end{array} & \left[\begin{array}{cc} b-\frac{c}{2} & b-c\\ b & 0 \end{array}\right] \end{array}.$$ (5)

Again, dividing each element by *b* and setting $\rho =\frac{c}{b}$ we obtain the following equivalent payoff matrix:

$$\pi =\begin{array}{cc} & \begin{array}{cc} C & D \end{array}\\ \begin{array}{c} C\\ D \end{array} & \left[\begin{array}{cc} 1-\frac{\rho }{2} & 1-\rho \\ 1 & 0 \end{array}\right] \end{array},$$ (6)

where *ρ* ∈ (0, 1). It again follows immediately from the rank ordering of the elements of the payoff matrix 6 that the snowdrift game is in the hawk-dove class of symmetric 2 × 2 games.

For the snowdrift game with payoff matrix 6, the Nash equilibria are the pure strategies (*C*, *D*) and (*D*, *C*) and the mixed strategy $\left(\frac{1-\rho }{1-\frac{\rho }{2}},\frac{\frac{\rho }{2}}{1-\frac{\rho }{2}}\right)$, the latter of which is also the unique ESS. Eq 2 gives the evolutionary dynamics for the game to be $\dot{p}=p\left(1-p\right)\left[p\left(\frac{\rho }{2}-1\right)+1-\rho \right]$. The equilibrium points for the dynamics are *p*^⋆^ = 0, *p*^⋆^ = 1, and $p^{⋆}=\frac{1-\rho }{1-\frac{\rho }{2}}$. The latter internal equilibrium is asymptotically stable, while the former two boundary equilibria are unstable. Thus, a population of individuals playing the snowdrift game, starting from an initial fraction *p*_0_ ∈ (0, 1) of cooperators, will evolve towards the internal equilibrium state ($p^{⋆}=\frac{1-\rho }{1-\frac{\rho }{2}}$) in which cooperators and defectors coexist.

#### Sculling game

Games in the coordination class have typically received little attention as models of cooperation, although for notable exceptions to this tendency see [32–35]. Here we introduce the sculling game as an exemplar of a social dilemma in the coordination class of games, with the aim of using it as a model for certain types of cooperative behavior not described by the donation or snowdrift games. We introduce this game through the following story. Two individuals John and Bill are rowing in a double scull to get to their destination. Reaching their destination at all has a value of $\frac{b}{2}$ to each of them, where $b\in R_{+}$. If they reach their destination before a certain time—which will happen only if they both row—then they can attend a party they would like to go to which has an additional value $\frac{3b}{2}$ to each of them. It is assumed that if they do not reach their destination at all, then the benefit to both is 0. Furthermore, rowing is assumed to carry a cost $c\in R_{+}$ to the rower. Therefore, if both row they reach their destination on time and can go to the party, and thus each receives a benefit of 2*b* and pays a cost of *c* for rowing, giving each a payoff of 2*b* − *c*. If neither rows, they drift in the river and do not reach their destination—thus, neither receives any benefit nor pays any cost for rowing, and each obtains a payoff of 0. If one rows and the other does not then they will eventually reach their destination, but late for the party, giving them each a benefit of $\frac{b}{2}$. The rower will pay a cost *c* for rowing and so obtain a payoff of $\frac{b}{2}-c$, while the non-rower will pay no cost and thus get a payoff of $\frac{b}{2}$. We shall assume that the benefit *b* and cost *c* satisfy 3*c* > 2*b* > *c*. If we treat rowing as the cooperative strategy *C* and non-rowing as the defective strategy *D*, then we can model the scenario described above as a symmetric 2 × 2 game, called the *sculling game*, with the payoff matrix

$$\pi =\begin{array}{cc} & \begin{array}{cc} C & D \end{array}\\ \begin{array}{c} C\\ D \end{array} & \left[\begin{array}{cc} 2b-c & \frac{b}{2}-c\\ \frac{b}{2} & 0 \end{array}\right] \end{array}.$$ (7)

As before, dividing each element by *b* and setting $\rho =\frac{c}{b}$ we obtain the following equivalent payoff matrix:

$$\pi =\begin{array}{cc} & \begin{array}{cc} C & D \end{array}\\ \begin{array}{c} C\\ D \end{array} & \left[\begin{array}{cc} 2-\rho & \frac{1}{2}-\rho \\ \frac{1}{2} & 0 \end{array}\right] \end{array},$$ (8)

where $\rho \in (\frac{1}{2},\frac{3}{2})$. It once again follows directly from the rank ordering of the elements of the payoff matrix 8 that the sculling game is in the coordination class of symmetric 2 × 2 games.

For the sculling game with payoff matrix 8, the Nash equilibria are the pure strategies (*C*, *C*) and (*D*, *D*), which are also ESSs, and the mixed strategy $\left(\rho -\frac{1}{2},\frac{3}{2}-\rho \right)$. Eq 2 gives the evolutionary dynamics for the game to be $\dot{p}=p\left(1-p\right)\left[p+\frac{1}{2}-\rho \right]$. The equilibrium points for the dynamics are *p*^⋆^ = 0, *p*^⋆^ = 1, and $p^{⋆}=\rho -\frac{1}{2}$. The former two boundary equilibria are asymptotically stable, while the latter internal equilibrium is unstable. Thus, a population of individuals playing the sculling game, starting from an initial fraction $p_{0}\in \left(0,1\right)\backslash \left\{\rho -\frac{1}{2}\right\}$ of cooperators, will evolve towards the all-defector (*p*^⋆^ = 0) state if $p_{0}<\rho -\frac{1}{2}$, and towards the all-cooperator (*p*^⋆^ = 1) state if $p_{0}>\rho -\frac{1}{2}$. We note that the equilibrium (*C*, *C*) is payoff-dominant over (*D*, *D*), for all *ρ*. Moreover, (*C*, *C*) is risk-dominant for $\rho \in (\frac{1}{2},1)$, while (*D*, *D*) is risk-dominant for $\rho \in (1,\frac{3}{2})$. A coordination game in which one of the two equilibria is payoff dominant while the other is risk dominant is a stag hunt game [33]. Thus, for $\rho \in (1,\frac{3}{2})$ the sculling game is an exemplar of the stag hunt game.

### Individual-Based Model on Networks

In this section, we define a stochastic individual-based model which allows the evolutionary dynamics of symmetric 2 × 2 games to be studied for populations with complex interaction patterns among their members [19, 46, 48, 49]. Consider a population of *n* individuals, labeled by *i* = 1, …, *n*. In order to allow the possibility of complex population interactions, we identify the individuals in the population with the set of vertices in a network Γ. The structure of Γ determines which individuals in the population interact. To be precise, two networks are required to specify the evolutionary dynamics—an interaction network, which specifies whether two individuals in the population can interact by playing the game, and an updating network, which specifies that an individual in the population can update its strategy by comparing its state to the states only of those individuals adjacent to it. Here, for simplicity, we shall assume that the interaction and updating networks are the same, and denote them both by Γ. The set of neighbors of *i* ∈ Γ (i.e., the set of individuals adjacent to *i* in Γ) will be denoted by *N*(*i*). The degree *k*_*i*_ of vertex *i* is the number of vertices adjacent to *i*, and the mean degree of the vertices in Γ is defined to be $k=\frac{1}{n}\sum_{i=1}^{n}k_{i}$.

The individual-based model is defined as a stochastic process on the network Γ. We shall start with an initial population in which a fraction *p*_0_ of the individuals use strategy *C* and remainder use strategy *D*. Each iteration of the evolutionary dynamics consists of an asynchronous interaction/update round, which involves sampling the population *n* times with replacement.

Each interaction/update step is carried out as follows (cf. [46, 48]). First, in the interaction phase we pick at random an individual *i* ∈ Γ and a random neighbor *j* ∈ *N*(*i*). In addition, we pick a random neighbor *k* ∈ *N*(*i*) of *i* and a random neighbor *l* ∈ *N*(*j*) of *j*. We then determine the payoff that player *i* receives from interacting with player *k* and the payoff that player *j* receives from interacting with player *l*. That is, if *p*, *q*, *r*, and *s* denote the strategies of *i*, *j*, *k*, and *l* respectively, then the payoff *P*_*i*_ received by the focal individual *i* is *π*(*p*, *r*) and the payoff *P*_*j*_ received by the individual *j* is *π*(*q*, *s*), where *π* is the payoff matrix for the game under consideration. Second, in the update phase the probability that the focal individual *i* will inherit *j*’s strategy, *p*_*i* ← *j*_, is determined using the *Fermi* update rule [88–90] as

$$p_{i\leftarrow j}=\frac{1}{1+e^{-\beta (P_{j}-P_{i})}},$$ (9)

where the parameter *β* > 0 is the “selection strength” of the update rule. Repeating the interaction/update step *n* times constitutes one generation of the evolutionary dynamics.

We note that the results of our individual-based simulations (described in the next section) are robust to changes in the update rule. For example, in addition to employing the Fermi update rule given by eq 9, we have also simulated the individual-based model using the *replicator* update rule [46, 91], in which the probability *p*_*i* ← *j*_ that the focal individual *i* inherits individual *j*’s strategy is given by

$$p_{i\leftarrow j}=\left\{\begin{array}{cc} 0 & \;\text{if}\;P_{i}\geq P_{j}\\ \frac{P_{j}-P_{i}}{P_{\text{max}}-P_{\text{min}}} & \text{otherwise,} \end{array}\right.$$ (10)

where $P_{max}=\max_{u\in \Gamma }P_{u}$, and $P_{min}=\min_{u\in \Gamma }P_{u}$. We find that the evolutionary dynamics of the symmetric 2 × 2 games that we study here is essentially identical irrespective of which of these update rules we employ. The results presented in the next section are from simulations using the Fermi update rule.

## Results

In this section we present the results of individual-based simulations for the donation, snowdrift, and sculling games. For all three games the individual-based model described in the previous section was simulated using the Fermi update rule (see eq 9) on the following model networks [63–66]: random regular networks; networks with exponential degree distribution (constructed using the growing random network model [92]); scale-free networks (constructed using the Barabási-Albert preferential attachment scheme [64]); and scale-free networks with different clustering coefficient *C* (defined below), generated using the Holme-Kim model [93] and assortativity coefficient *r* (defined below), generated by applying the rewiring algorithm of [94] to a Barabási-Albert’s scale-free network. The network size was *n* = 10000 for all these model networks.

A common property of many real-world networks is that they have a non-zero clustering coefficient [63, 66]. The clustering coefficient of a network Γ measures the average probability that two neighbors of a vertex are themselves adjacent. The local clustering coefficient *C*_*i*_ of a vertex *i* ∈ Γ is defined to be [63, 66]

$$C_{i}=\frac{(\text{number}\;\text{of}\;\text{pairs}\;\text{of}\;\text{neighbors}\;\text{of}\;i\;\text{that}\;\text{are}\;\text{adjacent})}{\left(\text{number}\;\text{of}\;\text{pairs}\;\text{of}\;\text{neighbors}\;\text{of}\;i\right)}.$$

The global clustering coefficient *C* ∈ [0, 1] for the whole network is then defined as the mean of the local clustering coefficients *C*_*i*_[63, 66]:

$$C=\frac{1}{n}\sum_{i=1}^{n}C_{i}.$$

A network with *C* = 1 has maximal clustering, while one with *C* = 0 has no clustering at all.

Another common property of many real-world networks is that they possess some amount of assortativity or disassortativity [95]. Assortative networks have the tendency that high-degree vertices are connected to other high-degree vertices and low-degree vertices are connected to other low-degree ones. In contrast, in disassortative networks, high-degree vertices tend to be connected to low-degree vertices and vice versa. Social networks are often assortative, while biological and technological networks are usually disassortative [95].

The assortativity (or disassortativity) of a network Γ can be quantified by the coefficient of assortativity *r* ∈ [−1, 1], defined by [95]

$$r=\frac{\sum_{i,j=1}^{n}\left(A_{ij}-k_{i}k_{j}/2m\right)k_{i}k_{j}}{\sum_{i,j=1}^{n}\left(k_{i}\delta_{ij}-k_{i}k_{j}/2m\right)k_{i}k_{j}},$$

where *A* is the adjacency matrix of Γ, *k*_*i*_ is the degree of vertex *i* ∈ Γ, *δ*_*ij*_ is the Kronecker delta, and *m* is the number of edges in the network. Networks with *r* > 0 are assortative, while those with *r* < 0 are disassortative. Networks with *r* = 0 are neither assortative nor disassortative.

In addition to model networks, we have simulated the donation, snowdrift, and sculling games on the following empirical networks: the network Γ_hep_ of coauthorships between scientists who posted preprints on the High-Energy Theory E-Print Archive between January 1, 1995 and December 31, 1999 [96]; the network Γ_astro_ of coauthorships between scientists who posted preprints on the Astrophysics E-Print Archive between January 1, 1995 and December 31, 1999 [96]; the largest component, Γ_fb_, of the New Orleans Facebook network of user-to-user links [97]; and a snapshot of the structure of the Internet, Γ_internet_, at the level of autonomous systems, reconstructed from BGP tables posted by the University of Oregon Route Views Project [66]. The basic properties of these empirical networks are listed in Table 1.

**Table 1:** Basic properties of empirical networks. Number of vertices *n*, average degree *k*, clustering coefficient *C*, and assortativity coefficient *r*.

Network	*n*	*k*	*C*	*r*
Γ_hep_	4786	5.334	0.644	0.197
Γ_astro_	16910	23.186	0.675	0.2
Γ_fb_	12968	47.521	0.269	0.092
Γ_internet_	14966	5.404	0.363	-0.216

All simulations were carried out for 10000 generations, with the initial fraction *p*_0_ of cooperators being 0.5, and with the selection strength *β* for the Fermi update rule set to 1.0. The results presented here were obtained by averaging the data from 50 independent runs of the model.

It is very useful to have a unitary measure of the total degree of cooperation for the donation, snowdrift, and sculling games on a given network. We will refer to the measure of cooperation that we introduce here as the *cooperation index* Λ or Λ-index. Let *p*_*t*_(*ρ*) denote the fraction of cooperators in the population at generation *t* in a game with cost-to-benefit ratio parameter *ρ* ∈ (*A*, *B*). The long-term mean ${\bar{p}}_{\infty }\left(\rho \right)$ of *p*_*t*_(*ρ*) is defined by

$${\bar{p}}_{\infty }\left(\rho \right)=\lim_{T\to \infty }\frac{1}{T}\sum_{t=1}^{T}p_{t}\left(\rho \right),$$ (11)

and the Λ-index for the three games is defined by

$$\Lambda =\frac{1}{B-A}\int_{A}^{B}{\bar{p}}_{\infty }\left(\rho \right)d\rho .$$ (12)

The cooperation index Λ ∈ [0, 1], where Λ = 1 represents complete cooperation, while Λ = 0 represents total lack of cooperation. The larger the value of Λ, the more cooperative the system. Thus, the Λ-index serves as a quantitative measure for comparing the totality of long-term cooperation in the population across the different networks. It is informative to compare the results we obtain for the Λ-index on networks with those that can be calculated analytically for the three games from the replicator dynamics in a large well-mixed population, which are as follows: for the donation game, ${\bar{p}}_{\infty }\left(\rho \right)=0$, *A* = 0, and *B* = 1, so Λ = 0; for the snowdrift game, ${\bar{p}}_{\infty }\left(\rho \right)=\frac{1-\rho }{1-\frac{\rho }{2}}$, *A* = 0, and *B* = 1, so Λ = 2 − ln 4 ≈ 0.614; and for the sculling game with initial fraction of cooperators $p_{0}=\frac{1}{2}$, ${\bar{p}}_{\infty }\left(\rho \right)=1$ if *ρ* < 1 and 0 otherwise, $A=\frac{1}{2}$, and $B=\frac{3}{2}$, so $\Lambda =\frac{1}{2}$.

### Donation Game

**Fig 1:**
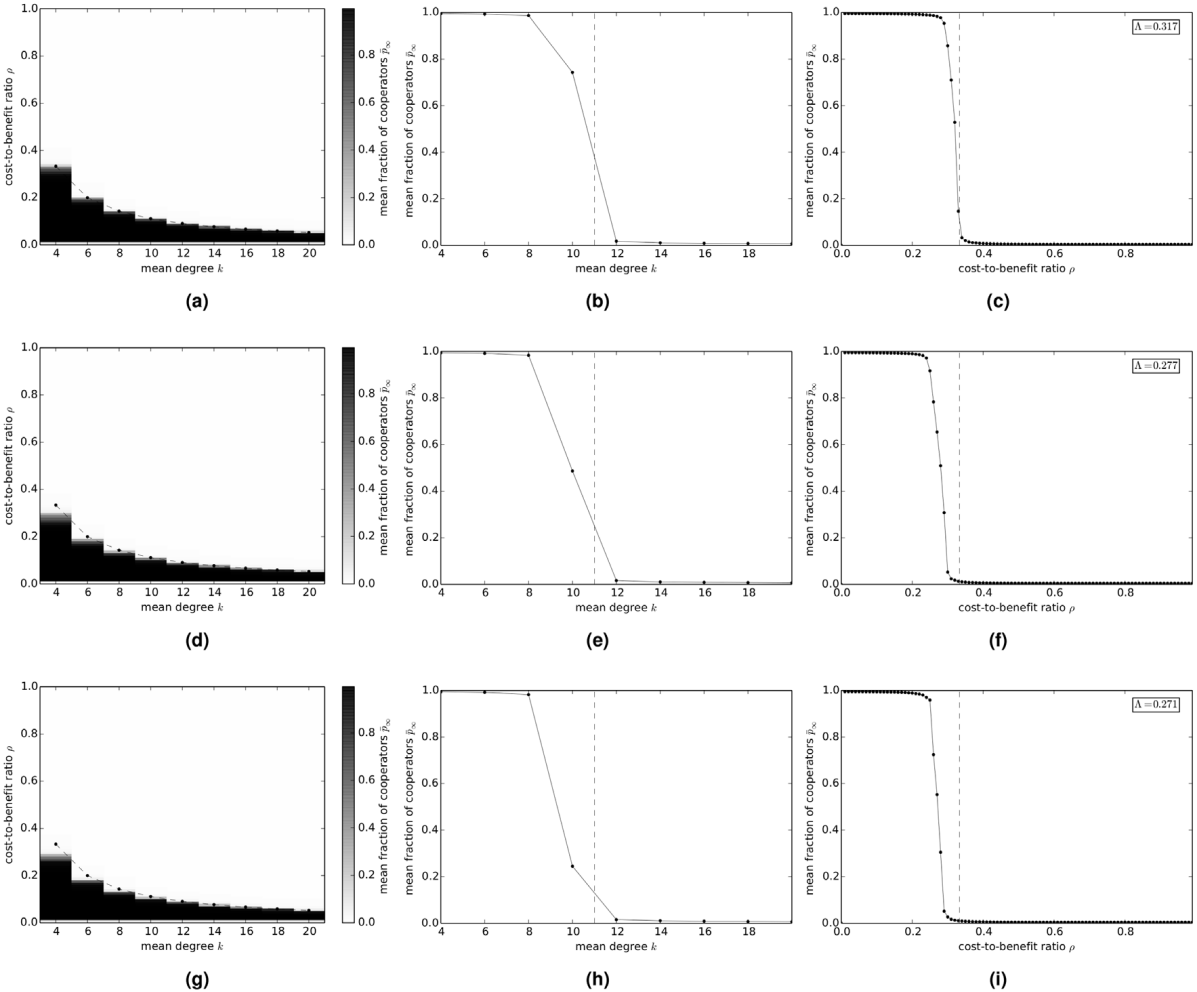
Variation of the long-term fraction ${\bar{p}}_{\infty }$ of cooperators with average degree *k* and cost-to-benefit ratio *ρ*, in the donation game on model networks. (a)-(c) random regular networks; (d)-(f) exponential networks; and (g)-(i) scale-free networks. (a)(d)(g) ${\bar{p}}_{\infty }$ versus *k* and *ρ*. The dashed lines show the graph of the relation $\frac{1}{\rho }=k-1$. (b)(e)(h) ${\bar{p}}_{\infty }$ versus *k* when *ρ* = 0.1. The dashed lines show the critical degree $k_{c}=\frac{1}{\rho }+1$. (d)(f)(i) ${\bar{p}}_{\infty }$ versus *ρ* when *k* = 4. The dashed lines show the critical value of the cost-to-benefit ratio $\rho_{c}=\frac{1}{k-1}$.

Fig 1(a)–1(c) shows the evolution of cooperation in the donation game on random regular networks. Fig 1(a) shows the variation of the long-term fraction ${\bar{p}}_{\infty }$ of cooperators with average degree *k* and cost-to-benefit ratio *ρ*. In Fig 1(b) the variation of ${\bar{p}}_{\infty }$ with *k* is shown when *ρ* is fixed at 0.1. Fig 1(c) shows the variation of ${\bar{p}}_{\infty }$ with *ρ* when *k* is fixed at 4—the inset in the figure shows the value of the cooperation index Λ. Fig 1(d)–1(f) and 1(g)–(i) show the corresponding results for the donation game on exponential networks and scale-free networks, respectively.

These results indicate that, for all three types of networks, as the average degree *k* of the network and/or the cost-to-benefit ratio *ρ* increases ${\bar{p}}_{\infty }$ undergoes a rapid transition from the totally cooperative state (${\bar{p}}_{\infty }=1$) to the totally defective state (${\bar{p}}_{\infty }=0$). The nature of the transition appears to be reasonably similar for all three network types. We observe that the transition from cooperation to defection appears to be governed to a good approximation by the condition that cooperation is favored only if $\frac{1}{\rho }>k-1$ (indicated by a dashed line in Fig 1(a), 1(d) and 1(g)). It follows from this condition that, for given *ρ*, cooperation only prevails on networks of average degree less than the critical value $k_{c}=\frac{1}{\rho }+1$ (indicated by a dashed line in Fig 1(b), 1(e) and 1(h)), and, for networks of given average degree *k*, cooperation only prevails for *ρ* less than the critical value $\rho_{c}=\frac{1}{k-1}$ (indicated by a dashed line in Fig 1(c), 1(f) and 1(i)). It further follows from the latter result that the Λ-index on a network of average degree *k* is approximately given by $\Lambda =\frac{1}{k-1}$, which is in reasonably good agreement with the simulation results.

These results imply that, for fixed *ρ*, as the average degree of a network increases, ${\bar{p}}_{\infty }$ transitions from the all-cooperator (${\bar{p}}_{\infty }=1$) state to the all-defector (${\bar{p}}_{\infty }=0$) state, which is reasonable since a network with a large average degree approximates the complete network, which represents a well-mixed population, for which ${\bar{p}}_{\infty }=0$. We note that the transition from total cooperation to total defection is sharpest for random regular networks and slightly less sharp for exponential and scale-free networks. We also note that the cooperation index Λ is highest for random regular networks and lowest for scale-free network, with exponential networks having an intermediate value of Λ.

**Fig 2:**
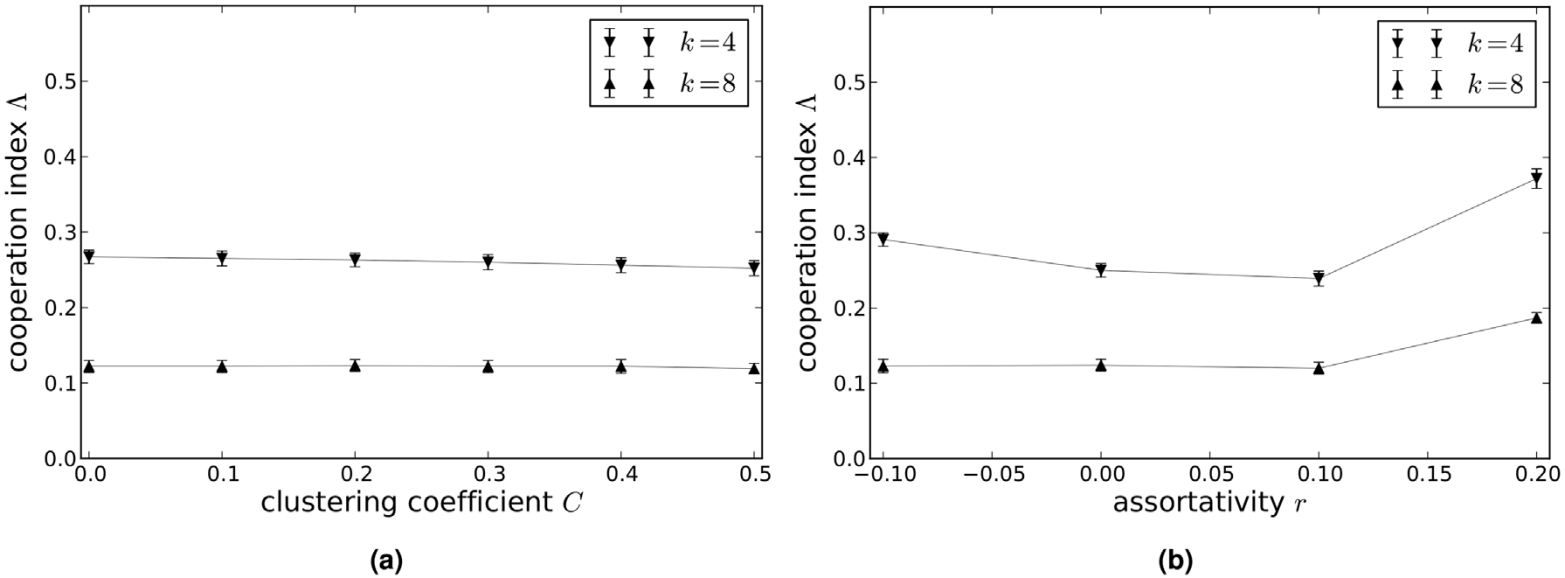
Values of the cooperation index Λ in the donation game on scale-free networks with clustering and assortativity. (a) Networks with clustering coefficient *C* = 0.0, 0.1, 0.2, 0.3, 0.4, 0.5. (b) Networks with assortativity coefficient *r* = −0.1, 0.0, 0.1, 0.2 (b). The values of Λ shown are the mean values calculated from 50 independent runs of the model, and the error bars (which are approximately the same size as the point markers) show the variance in Λ.

Fig 2(a) and 2(b) shows how the cooperation index Λ on scale-free networks with average degrees *k* = 4 and *k* = 8 varies with clustering coefficient *C* (a) and assortativity coefficient *r* (b). These results suggest that Λ is more or less unaffected by clustering, whereas Λ is higher for significantly assortative networks than for networks that exhibit little to no assortativity. For networks of average degree 4 there is also a slight increase in Λ for disassortative networks compared to those with little or no assortativity.

**Fig 3:**
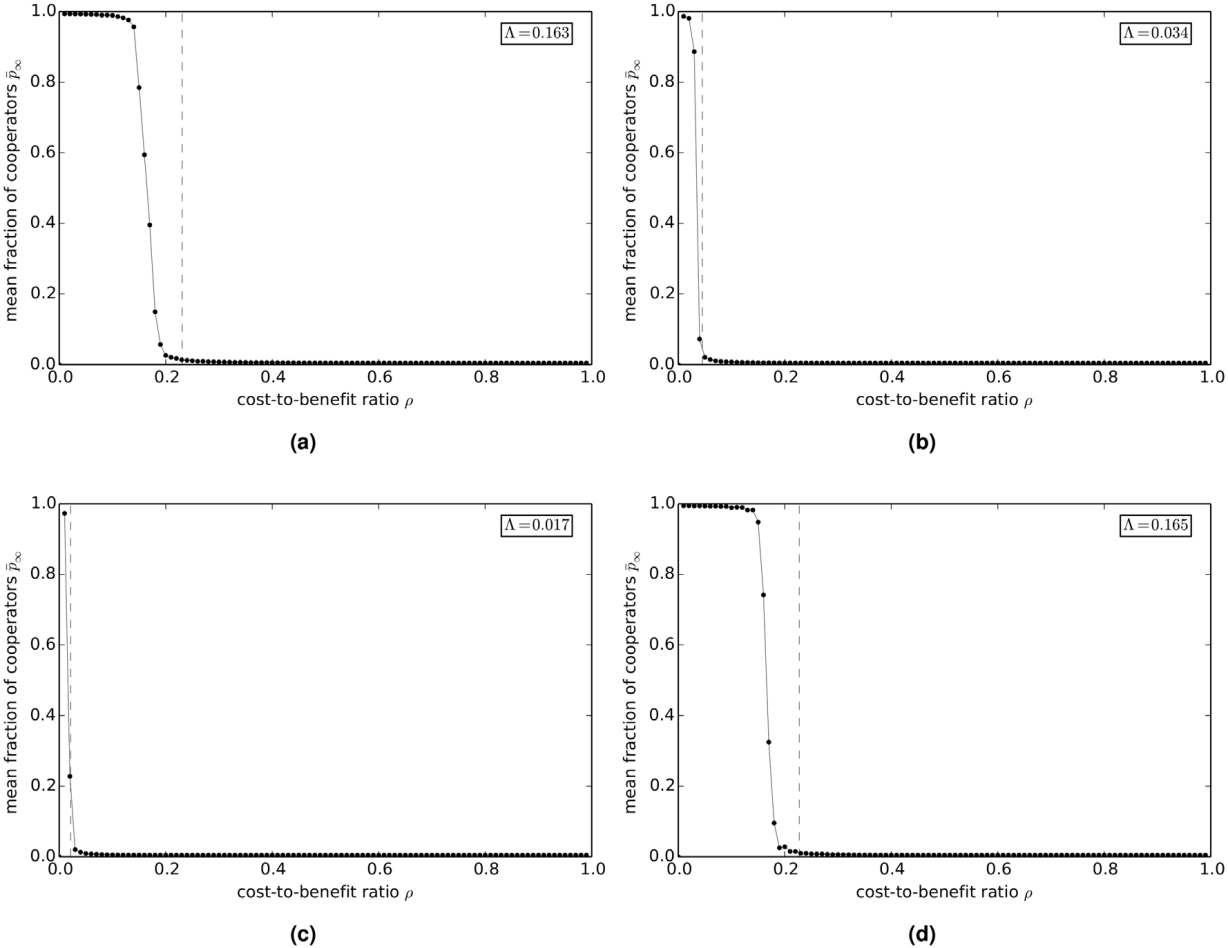
Long-term value ${\bar{p}}_{\infty }$ of the fraction of cooperators versus the cost-to-benefit ratio *ρ* in the donation game on empirical networks. (a) Γ_hepth_. (b) Γ_astro_. (c) Γ_fb_. (d) Γ_internet_. The dashed lines show the critical values of the cost-to-benefit ratio $\rho_{c}=\frac{1}{k-1}$.

Fig 3(a)–3(d) show the variation of ${\bar{p}}_{\infty }$ with *ρ* on empirical networks: Γ_hepth_ (a), Γ_astro_ (b), Γ_fb_ (c), and Γ_internet_ (d). The inset in the figures shows the value of the cooperation index Λ. The Γ_hepth_ and Γ_internet_ networks both have appreciable values of Λ, whereas for both the Γ_astro_ and Γ_fb_ networks Λ is small. The transition from cooperation to defection on these empirical networks appears also to be governed to a reasonable approximation by the same condition that was found to hold for model networks, namely cooperation is favored only if $\frac{1}{\rho }>k-1$ (indicated by a dashed line in the figures). Since it then follows that Λ is given approximately by $\Lambda =\frac{1}{k-1}$, the higher values of Λ found for Γ_hepth_ and Γ_internet_ and the lower values of Λ found for Γ_astro_ and Γ_fb_ are consistent with the results that would be expected given the lower average degrees of the former two networks and the higher average degrees of the latter two networks.

### Snowdrift Game

**Fig 4:**
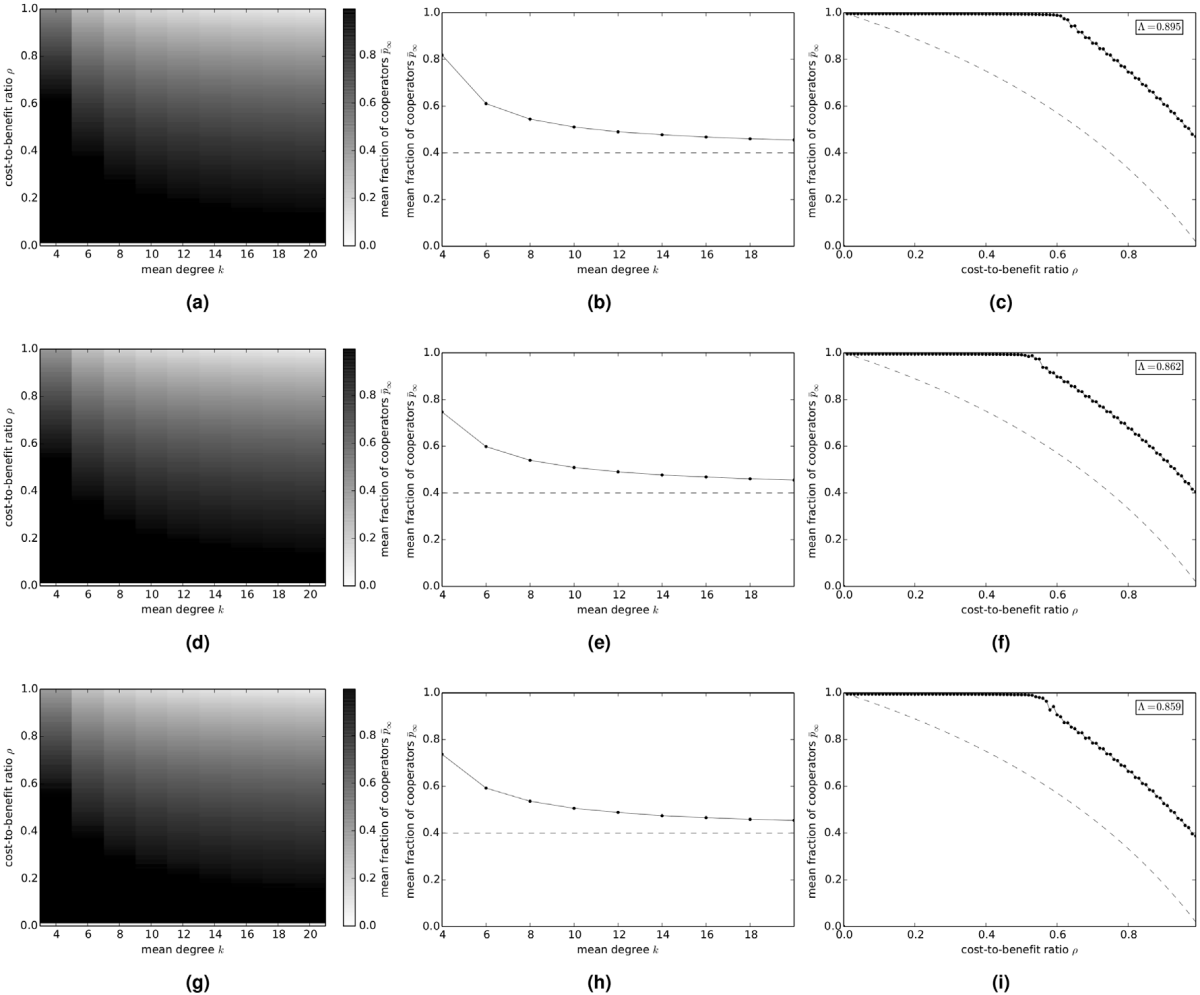
Variation of the long-term fraction ${\bar{p}}_{\infty }$ of cooperators with average degree *k* and cost-to-benefit ratio *ρ*, in the snowdrift game on model networks. (a)-(c) random regular networks; (d)-(f) exponential networks; and (g)-(i) scale-free networks. (a)(d)(g) ${\bar{p}}_{\infty }$ versus *k* and *ρ*. (b)(e)(h) ${\bar{p}}_{\infty }$ versus *k* when *ρ* = 0.75. The dashed lines show the asymptotic frequency of cooperators in a well-mixed population. (d)(f)(i) ${\bar{p}}_{\infty }$ versus *ρ* when *k* = 4. The dashed lines show the asymptotic frequency of cooperators in a well-mixed population.

Fig 4(a)–4(c) shows the evolution of cooperation in the snowdrift game on random regular networks. Fig 4(a) shows the variation of the long-term fraction ${\bar{p}}_{\infty }$ of cooperators with average degree *k* and cost-to-benefit ratio *ρ*. Fig 4(b) shows the variation of ${\bar{p}}_{\infty }$ with *k* when *ρ* is fixed at 0.75. Fig 4(c) shows the variation of ${\bar{p}}_{\infty }$ with *ρ* when *k* is fixed at 4—the inset in the figure shows the value of the cooperation index Λ, and the dashed line shows the variation of ${\bar{p}}_{\infty }$ with *ρ* in a well-mixed population. Fig 4(d)–4(f) and 4(g)–4(i) show the corresponding results on exponential networks and scale-free networks, respectively.

These results show that, for *ρ* = 0.75, ${\bar{p}}_{\infty }$ continuously decreases as the average degree of the network increases, approaching a value of 0.4 for *k* = 20 (indicated by a dashed line in Fig 4(b), 4(e) and 4(h)), which is reasonable because a network with a large average degree approximates the complete network, which represents a well-mixed population, for which ${\bar{p}}_{\infty }=0.4$. This behavior seems to be independent of the type of network. For a network with a given average degree *k*, ${\bar{p}}_{\infty }$ continuously decreases from higher values to lower values as the cost-to-benefit ratio *ρ* increases. The cooperation index Λ is not greatly affected by the type of network, although it is slightly higher on random regular networks and progressively lower on exponential and scale-free networks, respectively.

**Fig 5:**
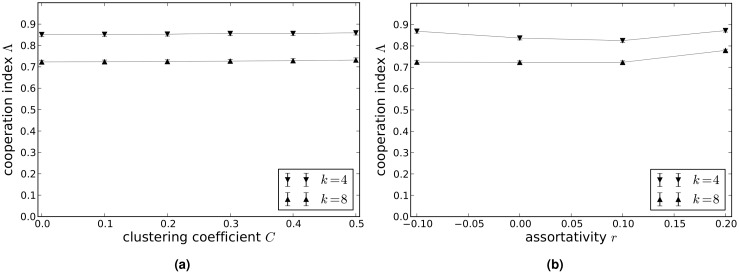
Values of the cooperation index Λ in the snowdrift game on scale-free networks with clustering and assortativity. (a) Networks with clustering coefficient *C* = 0.0, 0.1, 0.2, 0.3, 0.4, 0.5. (b) Networks with assortativity coefficient *r* = −0.1, 0.0, 0.1, 0.2. The values of Λ shown are the mean values calculated from 50 independent runs of the model, and the error bars (which are approximately the same size as the point markers) show the variance in Λ.

Fig 5(a) and 5(b) show how the cooperation index Λ on scale-free networks with average degrees *k* = 4 and *k* = 8 varies with clustering coefficient *C* (a) and assortativity coefficient *r* (b). Here again, Λ is more or less unaffected by clustering, whereas Λ is slightly higher for significantly diassortative and assortative networks than for networks that exhibit little to no assortativity. For networks of average degree 4 there is also a slight increase in Λ for disassortative networks compared to those with little or no assortativity.

**Fig 6:**
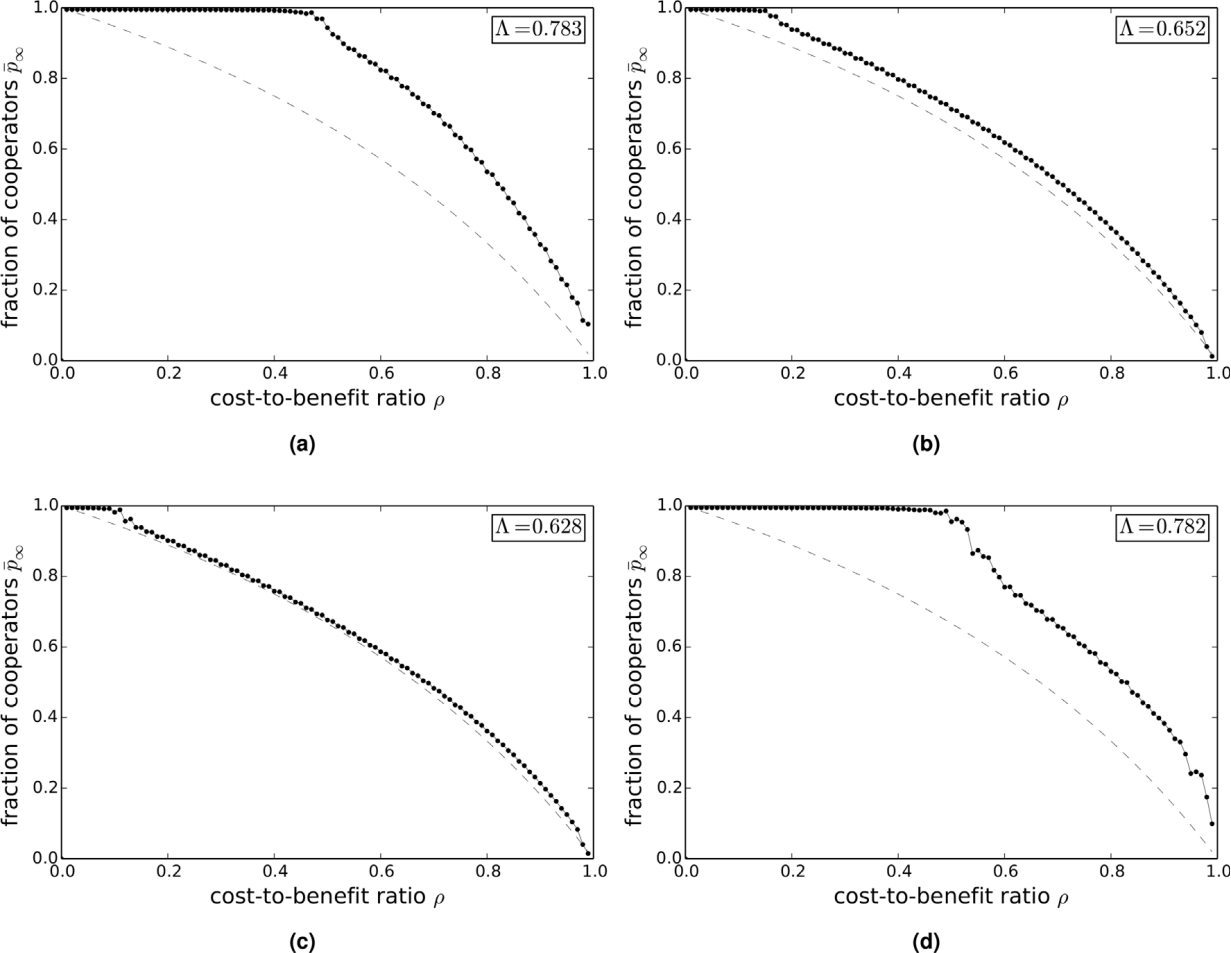
Long-term value ${\bar{p}}_{\infty }$ of the fraction of cooperators versus the cost-to-benefit ratio *ρ* in the snowdrift game on empirical networks. (a) Γ_hepth_. (b) Γ_astro_. (c) Γ_fb_. (d) Γ_internet_. The dashed lines show the asymptotic frequency of cooperators in a well-mixed population.

Fig 6(a)–6(d) shows the variation of ${\bar{p}}_{\infty }$ with *ρ* on empirical networks: Γ_hepth_ (a), Γ_astro_ (b), Γ_fb_ (c), and Γ_internet_ (d). The inset in the figures shows the value of the cooperation index Λ, and the dashed line shows the variation of ${\bar{p}}_{\infty }$ with *ρ* in a well-mixed population. In all cases the value of Λ is greater than the value Λ = 0.614 for a well-mixed population. The value of Λ for the Γ_hepth_ and Γ_internet_ networks is much higher than the value in a well-mixed population, while for the Γ_astro_ and Γ_fb_ networks, the value is only slightly higher than the value in a well-mixed population. These results are consistent with what would be expected given the differences in the mean degree of the various networks.

### Sculling Game

**Fig 7:**
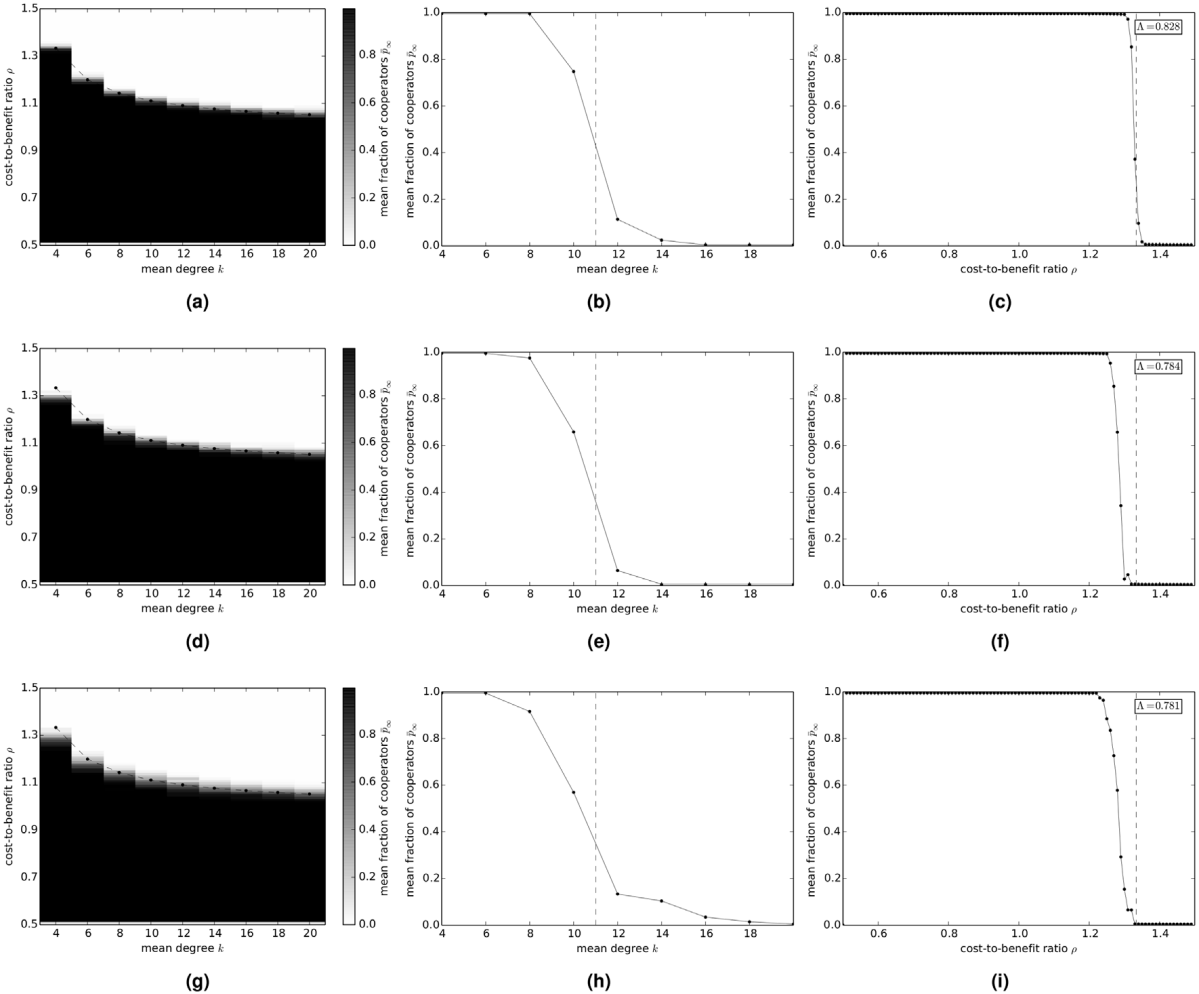
Variation of the long-term fraction ${\bar{p}}_{\infty }$ of cooperators with average degree *k* and cost-to-benefit ratio *ρ*, in the sculling game on model networks. (a)-(c) random regular networks; (d)-(f) exponential networks; and (g)-(i) scale-free networks. (a)(d)(g) ${\bar{p}}_{\infty }$ versus *k* and *ρ*. The dashed lines show the graph of the relation $\frac{1}{\rho -1}=k-1$. (b)(e)(h) ${\bar{p}}_{\infty }$ versus *k* when *ρ* = 1.1. The dashed lines show the critical degree $k_{c}=\frac{1}{\rho -1}+1$. (d)(f)(i) ${\bar{p}}_{\infty }$ versus *ρ* when *k* = 4. The dashed lines show the value of the critical cost-to-benefit ratio $\rho_{c}=\frac{1}{k-1}+1$.

Fig 7(a)–7(c) shows the evolution of cooperation in the sculling game on random regular networks. Fig 7(a) shows the variation of the long-term fraction ${\bar{p}}_{\infty }$ of cooperators with average degree *k* and cost-to-benefit ratio *ρ*. Fig 7(b) shows the variation of ${\bar{p}}_{\infty }$ with *k* when *ρ* is fixed at 1.1. Fig 7(c) shows the variation of ${\bar{p}}_{\infty }$ with *ρ* when *k* is fixed at 4—the inset in the figure shows the value of the cooperation index Λ. Fig 7(d)–7(f) and 7(g)–7(i) show the corresponding results on exponential networks and scale-free networks, respectively.

These results show that, for all three types of networks, as the average degree *k* of the network and/or the cost-to-benefit ratio *ρ* increases ${\bar{p}}_{\infty }$ undergoes a rapid transition from the totally cooperative state (${\bar{p}}_{\infty }=1$) to the totally defective state (${\bar{p}}_{\infty }=0$). The nature of the transition appears to be quite similar for all three network types. We observe that the transition from cooperation to defection appears to be governed to a good approximation by the condition that, for *ρ* > 1, cooperation is favored only if $\frac{1}{\rho -1}>k-1$ (indicated by a dashed line in Fig 7(a), 7(d) and 7(g)). We note that for *ρ* < 1, cooperation is both payoff dominant and risk dominant over defection and is thus favored on any network (including the complete network representing a well-mixed population). It follows from this condition that, for given *ρ* > 1, cooperation only prevails on networks of average degree less than the critical value $k_{c}=\frac{1}{\rho -1}+1$ (indicated by a dashed line in Fig 7(b), 7(e) and 7(h)), and, for networks of given average degree *k*, cooperation only prevails for *ρ* less than the critical value $\rho_{c}=\frac{1}{k-1}+1$ (indicated by a dashed line in Fig 1(c), 1(f) and 1(i)). It further follows from the latter result that the Λ-index on a network of average degree *k* is approximately given by $\Lambda =\frac{1}{k-1}+\frac{1}{2}$.

It follows from these results that, for fixed *ρ* > 1, as the average degree of a network increases, ${\bar{p}}_{\infty }$ transitions from the all-cooperator (${\bar{p}}_{\infty }=1$) state to the all-defector (${\bar{p}}_{\infty }=0$) state, which is reasonable since a network with a large average degree approximates the complete network, which represents a well-mixed population, which for *ρ* > 1 has ${\bar{p}}_{\infty }=0$. We note that, as for the donation game, the transition from total cooperation to total defection is sharpest for random regular networks and slightly less sharp for exponential and scale-free networks. We also note that, as for the donation and snowdrift games, the cooperation index Λ is highest for random regular networks and lowest for scale-free network, with exponential networks having an intermediate value of Λ.

**Fig 8:**
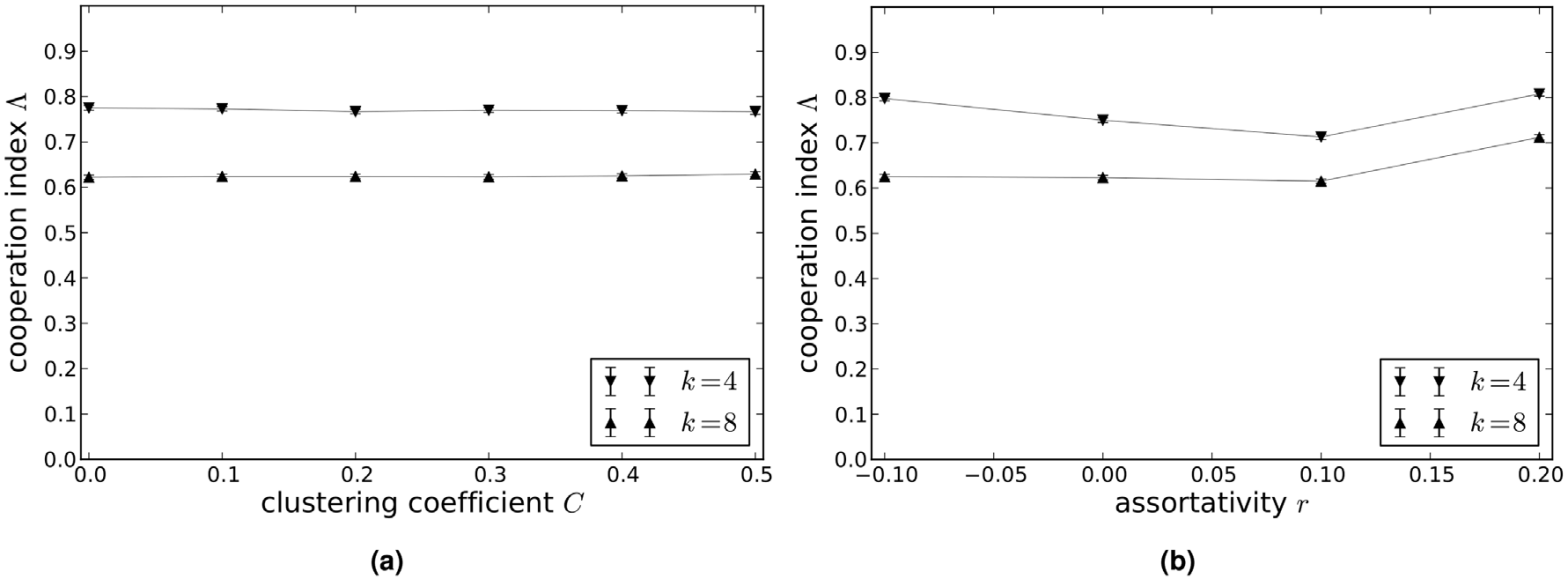
Values of the cooperation index Λ in the sculling game on scale-free networks with clustering and assortativity. (a) Networks with clustering coefficient *C* = 0.0, 0.1, 0.2, 0.3, 0.4, 0.5. (b) Networks with assortativity coefficient *r* = −0.1, 0.0, 0.1, 0.2. The values of Λ shown are the mean values calculated from 50 independent runs of the model, and the error bars (which are approximately the same size as the point markers) show the variance in Λ.

Fig 8(a) and 8(b) show how the cooperation index Λ on scale-free networks with average degrees *k* = 4 and *k* = 8 varies with clustering coefficient *C* (a) and assortativity coefficient *r* (b). As in the case of the donation and snowdrift games, Λ is more or less unaffected by clustering, whereas Λ is higher for significantly assortative networks than for networks that exhibit little to no assortativity. For networks of average degree 4 there is also a slight increase in Λ for disassortative networks compared to those with little or no assortativity.

**Fig 9:**
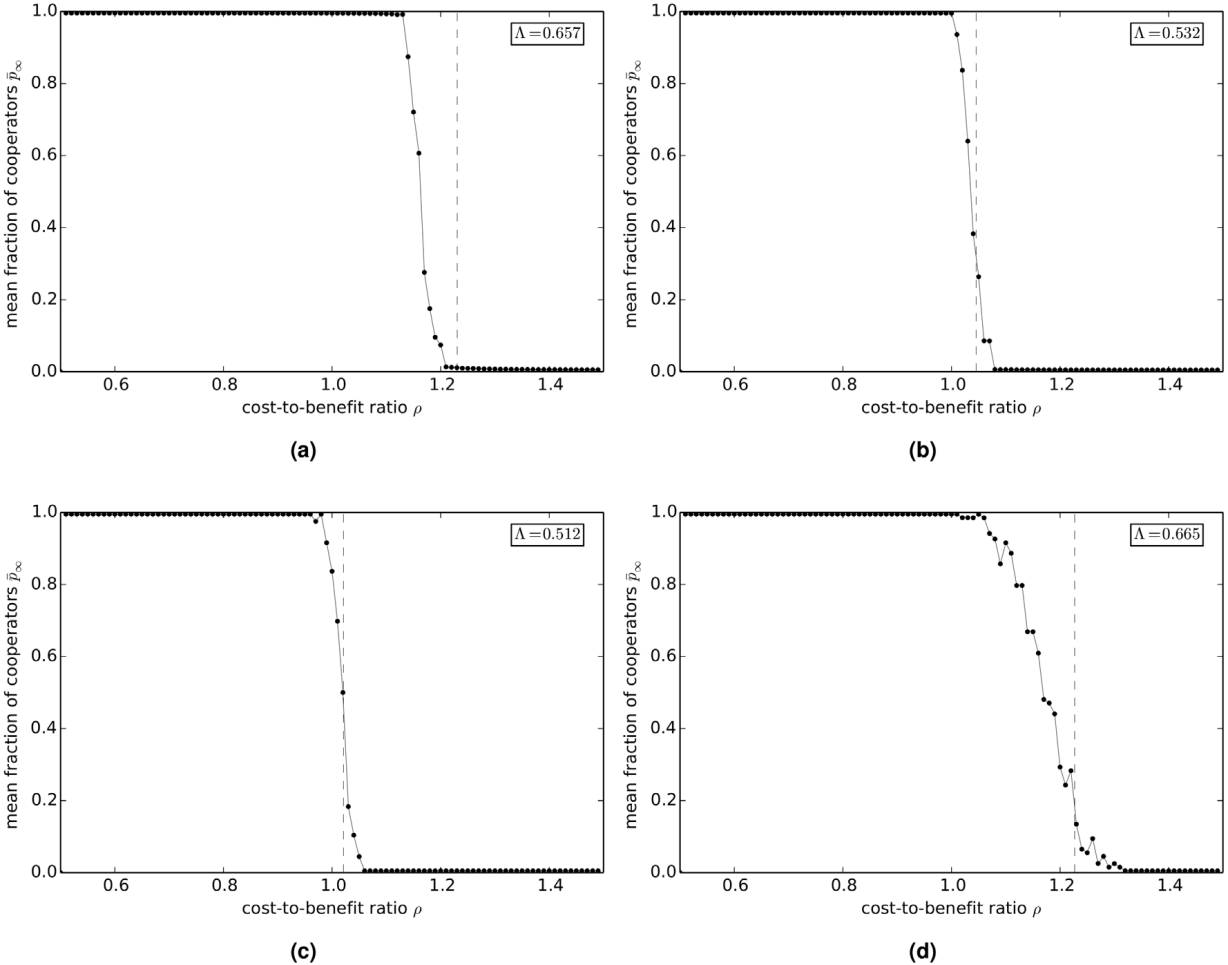
Long-term value ${\bar{p}}_{\infty }$ of the fraction of cooperators versus the cost-to-benefit ratio *ρ* in the sculling game on empirical networks. (a) Γ_hepth_. (b) Γ_astro_. (c) Γ_fb_. (d) Γ_internet_. The dashed lines show the critical values of the cost-to-benefit ratio $\rho_{c}=\frac{1}{k-1}+1$.

Fig 9(a)–9(d) show the variation of ${\bar{p}}_{\infty }$ with *ρ* on empirical networks: Γ_hepth_ (a), Γ_astro_ (b), Γ_fb_ (c), and Γ_internet_ (d). The inset in the figures shows the value of the cooperation index Λ. The value of Λ for the Γ_hep_ and Γ_internet_ networks is significantly greater than the value in a well-mixed population, while the value of Λ for the Γ_astro_ and Γ_fb_ networks is only slightly higher than the value in a well-mixed population. The transition from cooperation to defection on these empirical networks appears also to be governed to a reasonable approximation by the same condition that was found to hold for model networks, namely cooperation is only favored, for *ρ* > 1, if $\frac{1}{\rho -1}>k-1$ (indicated by a dashed line in the figures). Since it follows from this relation that Λ is given approximately by $\Lambda =\frac{1}{k-1}+\frac{1}{2}$, the higher values of Λ found for Γ_hepth_ and Γ_internet_ and the lower values of Λ found for Γ_astro_ and Γ_fb_ are consistent with the results that would be expected given the lower average degrees of the former two networks and the higher average degrees of the latter two networks.

## Discussion

In this paper we have studied the evolution of cooperation in social dilemmas in network-structured populations. The social dilemmas that we have focused on—the donation, snowdrift, and sculling games—are exemplars of the fundamental types of social dilemmas represented by symmetric 2 × 2 games, namely, the prisoner’s dilemma, hawk-dove, and coordination classes of games. Here we have investigated the effect of the structure of the network on which the population is modeled on the maintenance of cooperative behavior in each of these three exemplars of social dilemmas. This study yields several conclusions that extend previously known results in a number of significant directions. Interestingly, the results we obtain concerning the influence of network structure on the evolution of cooperation in each of the donation, snowdrift, and sculling games are all in good overall agreement and allow a number of general conclusions to be drawn. The most basic conclusion is that for all these social dilemmas the propensity for cooperation is increased on complex networks compared to a well-mixed population. This conclusion is generally well accepted for the donation game and other forms of the prisoner’s dilemma in network-structured populations [42, 43, 45, 47–49, 56]. For the case of the snowdrift game, this conclusion is consistent with the results found in [48, 98], and the inhibiting effect of spatial structure found in [46] appears to be limited to lattice networks. The evolution of cooperation in games of coordination class on networks has received relatively little attention, with the interesting exceptions of [32, 34, 35], and this paucity has motivated our introduction and study of the sculling game. Again for this game we find that network structure promotes the evolution of cooperation compared to a well-mixed population.

For the donation, snowdrift, and sculling games on networks the most basic network characteristic that affects the evolution of cooperation is the average degree of the network. For all these social dilemmas the level of cooperation decreases monotonically as the average degree of the network increases. This behavior is of course completely reasonable since as the average degree increases the network approximates more and more closely to the complete network, which represents a well-mixed population. For the donation and sculling games there are reasonably sharp transitions from cooperation to defection determined by the average degree *k* and the cost-to-benefit ratio *ρ*. In both cases the transition from cooperation to defection is apparently governed to a good approximation by simple relations—for the donation game cooperation only prevails if $\frac{1}{\rho }>k-1$ (with defection dominating otherwise) and for the sculling game cooperation only prevails, for *ρ* > 1, if $\frac{1}{\rho -1}>k-1$ (with defection dominating otherwise). These relations hold to a good approximation on a variety of model networks and also to a reasonable approximation on a range of empirical networks. We observe that among model networks the relations are most exactly obeyed for both games on random regular networks and least exactly obeyed on scale-free networks, which suggests that the accuracy with which these relations govern the evolution of cooperation in both games decreases as the variance in the degree distribution of the network increases. It is also the case that the overall level of cooperation in the donation and sculling games, as measured by the Λ-index, decreases as the variance of the degree distribution of the network increases. The relation we find governing the evolution of cooperation in the donation game (i.e., $\frac{1}{\rho }>k-1$) is clearly analogous to those found in [49, 98] for different update procedures. For example, it was found in [49, 98] that cooperation is favored with death-birth updating only if $\frac{1}{\rho }>k$ and with imitation updating only if $\frac{1}{\rho }>k+2$. Thus, we note that the Fermi update procedure we have used favors the evolution of cooperation at lower values of the average degree than the death-birth or imitation updating used in [49, 98]. The evolution of cooperation in the snowdrift game on complex networks does not display the sharp transition from cooperation to defection found in the donation and sculling games, but the same general pattern of decreasing cooperation with increasing *k* and/or *ρ* still holds, as does the decrease in cooperation as the variance in the degree distribution increases.

The general form of the dependence of the level of cooperation in all three games on the mean degree of the network can be understood by recognizing that the key determinant of the level of cooperation that is maintained in a game on a network is the extent to which the network structure results in assortative interactions occurring between the strategies used by the individuals in the population—that is, the extent to which the network structure results in individuals interacting more often with their own strategy type than would occur in a randomly interacting population. It is clear that the degree to which assortative interactions will be induced in a network structured population will increase as the average number of neighbors in the population decreases. Thus, the decrease in the levels of cooperation in all three games with increasing mean network degree is a consequence of the decreasing degree of strategic assortativity that arises as the average degree increases. This point of view also suggests a possible explanation for our finding that for all three games the level of cooperation that evolves decreases as the heterogeneity of the degree distribution of the network increases. Networks with highly heterogeneous degree distributions will have some vertices with very large degrees, and by definition these high degree vertices will be the neighbors of many other vertices in the network. However, high degree vertices will have low levels of assortative interactions which will result in them generally being less able to support cooperative strategies, and this in turn implies that many other vertices in the network will have such uncooperative individuals as their neighbors, which will inhibit the formation of clusters of cooperators and thereby reduce the overall level of cooperation.

We have also investigated the effect of the network properties of clustering and assortativity on the maintenance of cooperation in the donation, snowdrift and sculling games. The results results are again largely consistent for all three games and allow some general conclusions to be drawn. One very clear finding is that for all three social dilemmas the clustering coefficient of the network has no appreciable effect on the level of cooperation that is maintained. This is at first sight surprising as it might seem *a priori* that the increased local density of short closed paths that exist in networks with higher clustering coefficient could facilitate the emergence of local clusters of cooperators and thus increase the level of cooperation in such networks [56, 99]. A possible explanation for the result that an increased clustering coefficient does not lead to an increase in cooperation may be that while an increased clustering coefficient results in a high local density of short closed paths it necessarily also results in there being fewer edges between these dense local clusters [100], and this sparsity of connecting edges may inhibit the formation of large clusters of cooperators which are necessary to produce high levels of assortative interactions, thereby counteracting at a global level what may be a positive local effect of network clustering. The further study of the mechanisms through which clustering affects the evolution of cooperation in these three games seems to be an interesting topic for future research.

Another rather clear conclusion that we can draw from this study is that higher network assortativity results in higher levels of cooperation in all three games. A possible explanation of this phenomenon is suggested by the relation between the degree of vertices and the extent to which they can support assortative strategic interactions that was mentioned above. In networks that are degree assortative low degree vertices are preferentially connected to other low degree vertices. This results in a connected cluster of low degree vertices, which by this very fact, is able to maintain high levels of assortative strategic interactions, and therefore also maintain high levels of cooperation. It is interesting to note that according to the point of view that we are advocating, the evolution of cooperation in a network structured population is driven by the low degree vertices, and therefore mechanisms that serve to segregate the low degree vertices may be anticipated to have a positive effect on the level of cooperation that can be maintained. The positive effect of network assortativity on the level of cooperation that evolves in all three social dilemmas in network structured populations is intriguing in view of the fact that social networks are almost always significantly assortative. It appears, therefore, that real-world social networks possess an important structural property which promotes the level of cooperation that evolves in social dilemmas on these networks. While it may be that the underlying causes of assortativity in empirical social networks are unrelated to the effect that it has on the evolution of cooperation, it is nevertheless interesting to speculate that an important causative factor may be between group selection acting on social dilemmas in network structured populations, which favors those populations with higher levels of cooperation, and thus also favors those social networks with higher assortativity. The further investigation of the relation between network assortativity and the evolution of cooperation in social dilemmas in network structured populations is an interesting and potentially important topic for future study.

Obtaining analytical insights into the results we have established here using individual-based simulations seems to be a natural topic for further investigation. While it is beyond the scope of the present paper to discuss analytical methods in any detail it nevertheless seems worthwhile to comment briefly on possible analytical developments. First, it should be noted that no very general theoretical results are to be expected for frequency dependent systems, such as the social dilemmas we have studied here, on arbitrary networks [101]. However, it seems likely that analytical methods can be successfully applied to the problems studied here in special cases. In particular, it seems reasonable to anticipate that the conditions we have found that cooperation is favored in the donation game only if $\frac{1}{\rho -1}>k-1$ and cooperation is favored in the sculling game, for *ρ* > 1, only if $\frac{1}{\rho -1}>k-1$, can be established analytically in the weak selection limit *β* → 0 on Cayley trees using pair approximation techniques as in [49, 98]. This would require extending the pair approximation methods used in [49, 98] to apply to the form of the update rule used here. Obtaining analytical results for the dependence of the cooperation index Λ on the clustering coefficient or assortativity coefficient appears to be considerably more challenging theoretically. This is a consequence of the fact that networks with clustering or assortativity will contain many short closed paths which violate the key property required for the pair approximation to be accurate [66]. Thus, it seems likely that new analytical techniques will have to be developed in order to understand the dependance of the cooperation index on clustering and assortativity. The development of such methods may present an exciting challenge for future research.

Finally, we remark that a potentially important avenue for further research may be to understand at a deeper level the intriguing and unexpected parallel that has emerged between the donation game and the sculling game on networks, and to explain why the conditions for cooperation to evolve appear to be so similar for two games which are conceptually quite distinct.
